# Genetic testing—whether to allow complete freedom? Direct to consumer tests versus genetic tests for medical purposes

**DOI:** 10.1007/s13353-021-00670-z

**Published:** 2021-11-26

**Authors:** Madej Malgorzata, Sąsiadek Maria, Witt Michał

**Affiliations:** 1 Institute of Political Science, University of Wroclaw, Wrocław, Poland; 2 Department of Genetics, Medical University of Wroclaw, Wrocław, Poland; 3 Institute of Human Genetics, Polish Academy of Sciences, Poznan, Poland

**Keywords:** Direct-to-consumer tests, DTC, Legal acts, EU, Genetic medical tests, Genetic data protection

## Abstract

Direct-to-consumer tests opened the opportunity of genetic testing without medical supervision, e.g., without medical referral and medical interpretation of the results. Thus, these approaches allow for free access to information concerning individual genetic profile increasing the area of personal freedom, but also posing the risk of false (positive and negative) or misinterpreted results along with health and psychological negative consequences. The paper discusses medical and non-medical applications of DTC, exploring also the legal framework implemented by European states and organizations. These legal acts strive to control the developing DTC market through such basic principles as patient protection, informed consent, medical information confidentiality, and the rights to know and to refuse knowledge about one’s genetic predispositions.

Direct-to-consumer tests (DTC) are available in direct sale (in stores or on-line) and offer a possibility of genetic analysis, with no medical rationale and disregarding specialized, individualized interpretation of test results. DTC are not an element of genetic diagnostics in medical terms, nor do they provide a possibility of specialized interpretation of the results for medical purposes. They usually contain a kit to perform a test from saliva or other biological material along with instructions for consumer. The material is sent to the seller company for analysis.

The decision to perform such tests is made by clients willing to get information on their health or health risks, usually despite the lack of clinical indications for such an examination, as well as to obtain information on their origins, intellectual, psychological and sporting predispositions, the dynamics of an aging process, specific dietary requirements, and other genetically determined characteristics. The worldwide popularity of DTC results from technical ease combined with difficult access to medical genetic diagnostics, determined primarily by the insufficient number of clinical geneticists as well as high price of validated medical tests (Finney 2012; Horton et al. 2019). It should be stressed that DTC are applied not only in adults, but they are increasingly popular in pediatric population, too (Weissman et al. 2019).

The range of DTC tests varies among kits offered by different companies, but usually they cover mutations determining monogenic diseases (both dominantly and recessively inherited), variants modulating the individual risk of developing common diseases (hypertension, dementia, peptic ulcer, etc.), personality traits, pharmacogenetic tests (predicting response to certain drugs), and predictive tests to identify the risk of monogenic diseases in the offspring (Ayala-Lopez and Nichols 2020).

## Genetic counseling and tests for medical purposes

Genetic medical tests, performed for health purposes, are offered to patients with diagnosed or suspected genetic diseases. In such cases, genetic counseling and diagnosis are fully or partially (depending on the scope of medical services guaranteed by the insurer) financed and supervised by the insurance company. Such tests allow for the diagnosis of an existing disease, congenital defects, and/or intellectual disability syndromes and/or diseases that will develop during the patient’s life (e.g., in the case of late-onset diseases such as certain neurodegenerative diseases; pre-symptomatic diagnostics) (Zergollern-Ĉupak et al. 2014).

A separate group involves tests designed to identify heterozygotes in the population, i.e., to identify asymptomatic carriers of mutations causing recessively inherited diseases (people who have one mutated copy of the gene are healthy but can pass this copy of the gene on to their offspring—the disease occurs in those people who have two mutated copies of the gene). Such tests can be used in neonatal screening programs (the test will be applied to the entire population) or selectively to specific families or subgroups of the general population (Remec et al. 2021).

Genetic tests for medical purposes are performed in reference laboratories, subject to quality control both in terms of equipment, correctness of laboratory procedures, and professional qualifications of the staff. Such tests are performed both post- and prenatally. The prenatal tests could be carried out on the cells of the embryo as a part of the procedure of in vitro fertilization (pre-implantation diagnosis), which allows an assessment of the genetic alterations of the embryo and enables the parents to make an informed decision whether such an embryo would be implanted into the uterus or not. The genetic tests could also be performed on the fetal genetic material as part of prenatal diagnosis, which allows the parents to make an informed decision on continuing, or not, the pregnancy (Oh 2019).

Genetic diagnostics is also widely used in oncology, as it allows diagnosis of hereditary cancer syndromes (e.g., breast and/or ovarian cancer), precise diagnosis of the tumor (molecular classification of tumors, e.g., brain gliomas), determining the patient’s susceptibility to targeted treatment (use of optimally acting drugs in patients with a specific mutation in cancer cells; predictive tests), and establishing the prognosis of the disease (prognostic tests) (Kilbride and Bradbury 2020; Wang et al. 2021; Mandrell et al. 2021).

Pharmacogenetic tests are also quite commonly used to assess the individual, genetically determined biometabolism of specific drugs or groups of drugs thus to identify those who will optimally respond to given treatment/dose of given drug. In practice, this applies in particular to drugs used in cardiology and psychiatry (Dowd and Krause 2021).

The results of medical genetic tests performed for health purposes, together with a detailed analysis of pedigree and clinical data, form the basis for diagnosis of a disease and for genetic counseling not only for the patient but also for the whole family. This aspect is one of the elements which differentiate the scope of clinical genetics from the scope of patient care provided by specialists in other fields of medicine, whose medical activity concerns the patient and usually does not allow for identification of the disease/risk of disease in other members of their family. This regards relatives in the same generation and both ascendants and descendants (Zampatti et al. 2021).

Every type of genetic test, such as classical cytogenetics, molecular cytogenetics (array comparative genomic hybridization, aCGH, multiplex ligation-dependent probe amplification, MLPA, fluorescent in situ hybridization, FISH), or molecular methods (polymerase chain reaction, PCR, single nucleotide polymorphism, SNP) or genome sequencing with different methods, e.g., next generation sequencing (NGS), require precise laboratory interpretation by specialists in each of these techniques that is of cytogeneticists and/or molecular biologists. A genetic test performed incorrectly or misinterpreted at the laboratory may produce positive or negative false results causing dramatic consequences for both the patient and the family, who will then receive genetic counseling based on the incorrect genetic data.

Whole-genome analyses give an insight into genomes of examined individuals in its entirety, revealing different genetic variants: pathogenic, probably pathogenic, non-pathogenic, probably non-pathogenic, and variants of uncertain (or unknown) significance (VUS). The correct interpretation of the significance of genetic variants requires advanced knowledge, both in molecular biology and bioinformatics, and is based on the analysis of continuously updated bioinformatic databases containing information on all described and verified variants of the genome. For undescribed variants, the interpretation is based on the assessment of biological significance of the identified change, taking into account whether it is located within the gene, in the coding or non-coding part, whether this alteration changes the function of the gene, like, e.g., frame-shift mutations and codon STOP, or whether it is a variant probably non-pathogenic that is not leading to a change in the expression of the gene (Rehder et al. 2021).

The results of genetic tests are then interpreted by specialized clinical geneticists who, based on the patient’s clinical data, pedigree analysis, sometimes additional genetic tests of family members, analysis of genetic databases, and current literature, prepare personalized genetic counseling for the patient and her/his family. The results of genetic research developed in such a way constitute medical information in the legal sense, being the basis for clinical management, both therapy and prophylactic care (Franceschini et al. 2018).

The subject of many academic debates is the question of the special status of genetic testing against the full spectrum of laboratory medical research. Without taking a position on the issue of the genetic exceptionalism, it is necessary to emphasize the particular characteristics of genetic testing, including genomic testing, which significantly distinguish it from other medical tests. Above all, it is the fact that the result of a single genetic test performed in a patient with a genetic disease actually affects the entire family of the tested person and can potentially have far-reaching psycho-social and family effects, including discrimination and stigmatization. In addition, genetic testing is generally performed only once in a lifetime (the results are constant and unchanged) and generates sensitive personal data, which determines the requirement for enhanced security regime for processing such data (Horton and Lucassen 2019).

## Direct-to-consumer tests

From the formal point of view, DTC are not considered diagnostic tests; their results are not the basis of medical procedures and do not constitute an element of institutional health care. Therefore, they do not require medical justification for their application. Most clients are not familiar with the laboratories in which the tests are performed, nor do they have access to information concerning compliance with the procedures for performing and interpreting the results in these laboratories (Folkersen 2020; Thiebes 2020).

### DTC identifying mutations of documented importance in the etiology of human diseases

Among the tests of health significance, the primary example concerns the ones which identify mutations that determine high risk of genetic disorder, e.g., the test of genes generating high predisposition to cancer (*BRCA1* and *BRCA2* gene mutations).

The negative effects of such diagnostic procedures include the fact that identification of the mutation, without providing the patient with its appropriate interpretation, will not become a starting point for proper medical care, so it will not have a pro-health effect. Moreover, such piece of information, not supported by a qualified medical procedure, leads to negative medical, psychological, and social consequences. A special effect of performing tests identifying mutations in genes generating increased risk of cancer is, in the case of negative results (no mutation identified), gaining conviction of false safety interpreted as lower than average cancer risk, which may result in resignation from regular prophylactic tests or appropriate lifestyle. This creates a dangerous situation, as only 5–10% of all cancers develop as a result of an inherited mutation, and 85–90% of these conditions are sporadic. The absence of an inherited mutation only means that the patient does not have an increased risk of developing the cancer, but his or her risk is equal to the population risk.

The widespread availability of tests to identify mutations that determine high risk of developing serious diseases, in addition to numerous negative aspects, represents its positive side. Positive aspects include also an ability to identify carriers of mutations that pose a high health risk, despite the lack of pedigree data that would enable the patients to seek for medical care as well as other family members to perform the test. However, such diagnostics can only be effective if it is a part of a smoothly functioning referral system to a clinical genetics center for patients who tested positive (Oliveri 2018).

### DTC allowing for identification of gene variants of unclear clinical significance

DTC are also aimed at identifying genetic variants suspected of being relevant for multigenic diseases, with an unknown specific mechanism of genetic determination, and for multifactorial diseases, where environmental factors play a key role in addition to the not entirely clear genetic mechanism (e.g., celiac disease, most cases of Parkinson’s disease or Alzheimer’s disease). Interpretation of results of such tests is difficult and requires detailed knowledge of pedigree, clinical data, and information on environmental exposure to potentially pathogenic environmental factors. In the case of these diseases, however, even a detailed knowledge of pedigree and environmental factors along with the abovementioned genetic data does not allow for precise determination of the risk of the disease, giving merely a chance for counseling on the healthy lifestyle and prevention of specific exposure factors.

### Pre-conception DTC

Pre-conception DTC are designed to identify carriers of rare mutations in future parents, aimed at identification of an increased risk of giving birth to a child with a recessive genetic disease (currently more than 8000 such rare or ultra-rare diseases are known). These tests, supplementing a family genetic history analysis, can be requested by physicians as diagnostic genetic tests for persons suspected for carrying a pathogenic variant, e.g., cystic fibrosis, ß-thalassemia, or spinal muscular atrophy. In such families until a sick child is born, people planning to have offspring cannot feel assured that they are not carriers of such alterations. Particular ethical and clinical problems are associated with the risk of unsolicited findings, e.g., mutations leading to late-onset diseases (e.g., Huntington’s disease) or to an increased risk of developing cancer. Identification of rare or ultra-rare pathogenic variants of the genome in families not yet burdened with the birth of a child with a genetic defect allow the patients to make conscious procreative decisions: e.g., forego having biological children, perform targeted prenatal tests, choose in vitro fertilization with targeted pre-implantation diagnostics, or adoption of a sperm/oocyte. However, such procedures require specialist genetic counseling and highly specialized gynecological care. The condition for the effectiveness of this procedure is a decision on the most appropriate test covering as many pathogenic variants in a context of a particular population to which the client belongs (the need to take into account inter-population genetic heterogeneity). It is also important that the test is carried out by a certified laboratory and that the client with identified mutations is referred to a clinical geneticist who can interpret the result in clinical terms and then design the appropriate medical procedure.

A special aspect of this diagnosis is a false result: false positives have a chance to be corrected in the medical procedure, but false negatives give the client a misleading sense of security, in practice depriving them of proper medical care (Capalbo et al. 2021).

### Prenatal DTC

The development of testing fetal DNA circulating in maternal serum has opened a new era in the field of direct-to-consumer tests. Fetal DNA can be obtained in an entirely non-invasive way, by collecting solely a sample of maternal blood, avoiding invasive methods of collection material (trophoblast puncture, amnio-, or cordocentesis). The diagnostic range of tests performed on fetal DNA circulating in maternal blood serum is limited to detection of selected aberrations (e.g., trisomy of chromosomes 21, 13, and 18, ploidy of chromosomes X and Y and selected microdeletions) (Skirton 2015). Thus, the laboratory interpretation of the results is relatively easy and does not require advanced knowledge of genetics and molecular biology. The inclusion criteria for such tests are as follows: single pregnancy, risk of genetic defect in the fetus not higher than 1:100 and not lower than 1:1000, no anatomical defects of the fetus. They enable identification of fetuses with high risk of the most common aberrations and are the basis for referring the pregnant woman to specialist genetic and gynecological care (Familiari et al. 2021).

### Pharmacogenetic DTC

Pharmacogenetic tests aim to determine individual sensitivity to drugs, i.e., the balance between the effective and safe dose, resulting from genetically determined activity of enzymes involved in the metabolism of drugs. The most commonly tests concern sensitivity to targeted drugs used in oncology and to antidepressants or opioids used in psychiatry.

DTC tests for oncologic patients to predict response to targeted therapy based on genetic alterations in cancer cells are clinically relevant only if ordered by oncologists. This is due to the fact that the choice of oncological treatment depends not only on genetic changes but also on the clinical course of the disease (TNM grading, clinical condition of given patient, results of prior treatment, etc.) as well as availability of drugs in a given country. Therefore, only a professional therapist knows which genetic test is of practical importance for the treatment of an individual patient (Filipski et al. 2017).

### Lifestyle DTC

These tests are not designed to identify potentially pathogenic genetic lesions and therefore do not bring any information about the health of the client or their family. They are advertised in the consumer offer as tests to determine, e.g., intellectual and psychological predispositions, athletic abilities, dynamics of the aging process, propensity to obesity, specific dietary indications, and other characteristics caused by an interplay of environmental and genetic factors.

These tests are usually based on analysis of selected SNPs in particular genes in most of the cases being of undocumented importance for the trait under study. Thus, these tests have no significant importance for the client’s self-awareness of health. Most often, they have neither positive nor negative implications for the consumer.

## Significance of false-negative and false-positive DTC results

The fact of providing false-negative or false-positive results in the case of lifestyle tests has no significant impact on life and health of the client. However, false results pose a serious threat in the case of tests of clinical importance.

False positive stands for information on a genetic lesion that entails either the risk of contracting a specific disease or the risk of having a diseased child. False negative implies that the client receives false information on no risk of genetic disease. Both of these situations, if not properly counseled, may lead a client to make inappropriate life decisions, cause undue stress or give a false sense of security (Tandy-Connor et al. 2018).

DTC are usually performed by the analysis of single nucleotide polymorphisms (SNPs) using microarray technology. SNPs are the most common form of molecular variants; on average, there are 4–5 million SNPs in a single human genome. Currently, about 100 million SNPs have been identified in the human population. SNPs occur both in the coding part of the genome, affecting gene function, as well as in the non-coding part of the genome being a neutral, specific individual genetic markers. Most of SNPs are irrelevant to the health and development of the individual. Some of SNPs may be considered biological markers, for such traits as sensitivity to carcinogens or certain disease risk. A lot of research is currently underway to identify SNPs or SNP panels that can be used to identify people with a higher risk of multifactorial diseases such as heart disease, diabetes, or sporadic cancers.

The same SNPs may be present in many people, or only in some members of the population as “private” variants, thus suitable for identification of the origin of genetic material. This is used, e.g., in paternity testing and to identify human remains or to establish family history. DTC based on SNP analysis are constructed using data of genome wide association studies (GWAS). By 2017, more than 3200 GWAS studies described more than 55,000 SNPs possibly associated with over 3000 diseases. A comparison of the composition of large DTC diagnostic panels produced for the same purpose by three largest commercial companies (Ancestry, 23andMe, MyHeritage) showed that only 16–18% of SNPs used in each of the panels were identical (Kim 2019). Such significant differences indicate that each company determines its own composition of the set of SNPs tested. For most of these SNPs, there is no clearly established clinical relevance.

For diseases with multigenic/multifactorial etiology and diseases characterized by allelic and non-allelic heterogeneity, analysis of a single SNP is irrelevant. Except for the few diseases for which a change in a single SNP is critical (such as APOE4 variant in Alzheimer’s disease), for the vast majority of diseases, the odds ratio (OR; the ratio of the chance of a given event occurring in a given group as compared to the same event occurring in another group) for a single SNP is below 1.5, showing lack of association. For such diseases, the analysis should be based on the assessment of multiple SNPs at the same time (Tandy-Connor et al. 2018).

The scale of the risk of false DTC results is evidenced by studies in which the results of selected DTCs were further verified: about 40–50% of such studies were found to be false positive. Some companies offer their customers an opportunity to verify the changes identified in DTC through NGS sequencing (Tandy-Connor et al. 2018), which significantly increases the reliability of DTC tests.

False DTC results are usually due to several reasons:

1. The examination of single SNPs in a given gene does not constitute a true molecular diagnosis and, with the exception of just a few diseases (such as the APOE4 variant mentioned above), does not provide information about the consumer’s health either now or in the future. The vast majority of monogenic diseases are characterized by allelic heterogeneity (numerous mutations of a single gene, e.g., cystic fibrosis, caused by mutations of the *CFTR* gene, with about 2000 variants described in this gene) or nonallelic heterogeneity, which means that a given disease is caused by a mutation(-s) in one of several different genes (e.g., hearing loss).
2. An interesting example is the DTC test of the *BRCA1* gene, whose pathogenic mutations determine increase of risk of breast and ovarian cancer. Currently, there have been identified about 1.200–1.300 mutations in this gene. Only a fraction of these lesions is analyzed in DTC tests (usually founder mutations, which are the most common alterations in given population and were identified for, e.g., Ashkenazi Jews, Poles, Lithuanians, Bahamas, French-Canadians, and Duch). Such a selective analysis is a priori seriously flawed because it does not detect the existing pathogenic mutation of *BRCA1* in about 80% of cases. Thus, it is clear that without individualized genetic counseling, persons receiving a negative result may be given false reassurance that there is no genetic predisposition, which may only be due to the fact that the analysis did not cover all possible pathogenic lesions in this gene (Narod and Salmena 2011). There are differences between various human populations in the frequency and type of SNPs occurring. Therefore, a separate OR should be calculated for each SNPs in a particular DTC to assess the predictive significance of the tested SNPs in relation to a given disease. This should also take into account an ethnic origin of the tested person and the population in which the GWAS tests were conducted, which are the basis for the construction of the test (Tandy-Connor et al. 2018).

## Genetic data protection

The growing popularity of DTC offers and the carelessness with which some institutions, not necessarily involved in genetics, provide various types of genetic tests prompts a close look at the issue of data security. In the era of personalized medicine, whole-genome sequencing is increasingly offered or recommended as a routine procedure. Not everyone is aware of how such procedures should be secured from the point of view of data privacy and confidentiality, and what the availability of genetic counseling should be both before and after the procedure. This inappropriate approach is not at all uncommon, particularly when these procedures are offered by biological or chemical centers which have a different approach to diagnostic issues from that of centers specializing in genetic diagnosis. A separate problem is the use of diagnostic and sequencing services in foreign laboratories, particularly in Asia, whose practices are often far below the ethical standards in force in other parts of the world. In such situations, there is little control over the applications of biological material and genetic data. The key to national security in this area should be appropriate legislation that protects both the subjects and the medical professionals who use the relevant diagnostic tools.

Since genetic data are legally considered sensitive personal data, it must be assumed that the patient (family) has the right to expect keeping this data fully confidential both during and after completion of medical procedures, similarly to security of any other medical data. If the patient does not have confidence in the geneticist to keep all genetic data obtained during the diagnostic process secret, his/her willingness to provide information on his/her own and family matters is limited. For the geneticist, this implies that an extremely important tool of genetic diagnostics as compiling a complete health and family history might become unavailable. It is also possible that a geneticist who does not guarantee to keep medical confidentiality cannot count on the patients to report to him or her at all (Witt and Witt, 2016).

In practice, however, there are circumstances enabling violation of the confidentiality of genetic data. This is mainly related to disclosure of data gained during the diagnostic procedure, which are important for the health or life of the family members of the diagnosed person. It is obvious that not all genetic data are equally important, also in the context of informing third parties. This is due to various factors, like severity of a health problem—it would be difficult to imagine failure to contact a third party (family) in the case of a cancer or long QT syndrome (LQTS), which may pose a direct threat to the life of both the patient and their family members. A size of the genetic risk, possibility to reduce it or the availability of medical intervention, are also important factors. Cases of low genetic risk are handled differently from cases with a 50% or higher risk; a reduction in genetic risk may be achieved by making rational decisions about a lifestyle, procreation, application of assisted reproduction techniques, or prenatal diagnosis. Rarely, interventional measures are applied which may give permanent positive health effects, such as elimination diet in phenylketonuria or colectomy in familial adenomatous polyposis coli (Berliner, 2015).

A particular difficulty of the problem of genetic confidentiality is that none of these factors can be treated separately because neither operate in a void—all of them have to be evaluated at the same time, making the problem even more complex and difficult to solve. This illustrates the importance of expert genetic counseling, which should bring all these factors together into one consistent and comprehensible framework.

## Legal provisions on genetic testing

As early as 1997, the Council of Europe developed the Convention for the Protection of Human Rights and Dignity of the Human Being with regard to the Application of Biology and Medicine (Convention, 1997). The Convention, commonly referred to as the Oviedo Convention, or the European Bioethical (or Biomedical) Convention, or EBC for short, is the only international legal act comprehensively addressing the fundamental problems of modern biomedicine. The Convention belongs to the category of legal instruments constituting the so-called hard law (as opposed to the non-binding “soft law”: resolutions, codes of conduct, recommendations, etc.), the ratification of which creates specific legal obligations for the national legislator, becoming immediately one of the elements of the existing legal order of the ratifying country. The Convention has been signed by 35 states and ratified by 29 of them (Committee on Bioethics, 2019). Poland is one of those states which failed to ratify the Convention upon its signing.

The methodology of the Convention assumes the development of detailed regulations in particular fields through additional protocols, while the Convention itself defines the basic objectives and principles. It serves to always protect individuals, and imposes an obligation of obtaining patients’ informed consent, ensuring also special protection of those unable to provide informed consent. According to the Convention, access to medical services should be equal and rendered “*in accordance with relevant professional obligations and standards*.” The Convention provides also that knowledge of genetic characteristics and heritage must not be used for any discriminatory measures (Convention, 1997).

Although member-states of the European Union belong also to the Council of Europe, the EU has taken its own effort in regulating the issues of genetic testing, with the Directive on in vitro diagnostic medical devices of 1998 Directive 98/79/EC, 1998), soon to be replaced with the much more detailed Regulation on in vitro diagnostic medical devices (Regulation (EU) 2017/746). The Regulation which is directly binding for all member states enhances the system of standards for devices, but it also touches on the aspect of genetic testing as services, referring to the medical counseling and supervision as well as the problem of informed consent (Kalokairinou et al., 2018).

The purpose of proper protection of genetic data is addressed by the European Union’s regulation on the protection of personal data published in 2018 (*Official Journal of the European Union* L 119), which has quite precisely defined what the term genetic data signifies in a legal sense. The Regulation specifies that genetic data have the status of personal data which relate to human genetic characteristics, reveal unique information about physiology or health, and result from the analysis of a person’s biological sample. This definition broadly coincides with the specification of general medical data, but differs in terms of noting the uniqueness of the information, which is treated as a specific and exclusive attribute of genetic data solely. Although the document itself does not state it explicitly, it can be inferred that it also concerns the protection of data of unborn subjects (data obtained, e.g., from prenatal diagnosis), which seems to be an obvious intention of the legislator.

Apart from the international provisions, nineteen states in Europe have already implemented their “genetic laws,” in a variety of ways regulating issues relating to diagnostic and/or scientific research in the genetic field, e.g., the German *Gendiagnosikgesetz-GenDG*, which was enacted by the Bundestag in 2009 and which sets very strict standards for the performance of such tests in the Federal Republic of Germany, can serve as an excellent example. In most cases, these provisions refer to the subjects of medical supervision over the testing, genetic counseling for patients, and ensuring informed consent; however, other solutions refer also to data storage and collection, requirements for centers authorized to perform genetic testing (Hoxhaj et al., 2020). Elsewhere, genetic testing is covered by broader provisions on medical services. Also, the USA have the 2008 Genetic Information Nondiscrimination Act (GINA), a legal act that prohibits discrimination on the basis of genetic information with regard to health insurance and employment on the federal level, while state regulatory provisions are very diverse (Tamir 2010). In Poland, as a general rule, the overall framework of supervision of healthcare services should be applied; however, considering the variable forms and commercial nature of the DTC genetic tests, it does not sufficiently cover all cases and situations.

## Conclusion

Direct-to-consumer genetic testing is quite a relatively new issue, but at the same time a challenging one, as inappropriate application of these tests may lead to very serious and harmful, frequently irreversible consequences. It touches on fundamental human rights, including the right to identity and integrity, right to medical care, protection of personal data, and non-discrimination. It involves a dichotomy of the right to self-awareness and knowledge of one’s health and broadly discussed “right not to know.” At the same time, in view of rapid development of DTC services offered by commercial suppliers, including online services which may be rendered outside the patient’s country of residence, they become a dynamic market that is very difficult to control. This is why a well-considered system of provisions should be implemented on the national level, basically to regulate the current condition of the market, but also to forecast and address its potential future developments. Legal acts of the European Union and Council of Europe provide a good starting point to design such a system with the utmost purpose of assisting and protecting patients, while at the same time supporting scientific and social development.
